# Neurofilaments in blood is a new promising preclinical biomarker for the screening of natural scrapie in sheep

**DOI:** 10.1371/journal.pone.0226697

**Published:** 2019-12-19

**Authors:** Henrik Zetterberg, Elena Bozzetta, Alessandra Favole, Cristiano Corona, Maria Concetta Cavarretta, Francesco Ingravalle, Kaj Blennow, Maurizio Pocchiari, Daniela Meloni

**Affiliations:** 1 Clinical Neurochemistry Laboratory, Sahlgrenska University Hospital, Mölndal, Sweden; 2 Institute of Neuroscience and Physiology, Department of Psychiatry and Neurochemistry, The Sahlgrenska Academy at the University of Gothenburg, Mölndal, Sweden; 3 Department of Neurodegenerative Disease, UCL Institute of Neurology, Queen Square, London, United Kingdom; 4 UK Dementia Research Institute at UCL, University College London, London, United Kingdom; 5 Istituto zooprofilattico del Piemonte Liguria e Valle d’Aosta, Turin, Italy; 6 Department of Neuroscience, Istituto Superiore di Sanità, Roma, Italy; FDA, UNITED STATES

## Abstract

Scrapie is a fatal neurodegenerative disease of sheep and goats belonging to the group of Transmissible Spongiform Encephalopathy or prion diseases. The EU has adopted mandatory measures for scrapie surveillance to safeguard public and animal health because it is highly contagious and might decimate all genetic susceptible animals in affected flocks. Definite diagnosis of scrapie relies on the detection of the pathological prion protein in brain tissues and there are still no blood biomarkers available for making diagnosis in living animals that can be used for the screening of sheep in scrapie-affected flocks. Neurofilament light (NfL) protein, a valid biomarker for neuronal and axonal damages, can now be easily measured in blood by the ultra-sensitive single molecule array (Simoa) technology. Recent work reported that serum NfL is increased in neurodegenerative diseases, including human prion diseases, but no data are available for scrapie or other animal prion diseases. Here, we found that the median serum NfL concentration in scrapie animals (56.2, IQR 42.2–84.8, n = 9) was more than 15 times higher (p = 0.00084) than that found in control samples (3.4, IQR 3.0–26.3, n = 11). Moreover, serum NfL concentration in scrapie sheep with clinical signs (n = 2; 75.3, 15.7 pg/ml) did not significantly (p = 0.541; t-test) differ from scrapie animals without clinical signs (n = 7; 61.0, 10.7 pg/ml). The receiver operating characteristic (ROC) curve analysis estimated the cut-off value of 31 pg/ml serum NfL for distinguishing scrapie-infected sheep from controls. The application of this cut-off value gives an accuracy of the test of 95% (percent error of 5.23%). These data indicate that the Simoa test for serum NfL might be a useful screening method for detecting preclinical scrapie in living sheep. Finally, the preliminary data reported here need confirmation in large and more structured studies.

## Introduction

Transmissible spongiform encephalopathy (TSE) or prion diseases are a group of fatal progressive neurodegenerative pathologies of humans and animals. Bovine spongiform encephalopathy (BSE), scrapie of sheep and goats, and Creutzfeldt–Jakob disease (CJD) of humans are among the most notable TSE diseases [1]. BSE has been unequivocally linked to the appearance of variant CJD in humans, while scrapie, an endemic disease in Europe for >200 years, has never been convincingly associated with any form of human TSE diseases [2,3], apart from recent data on the experimental transmission of scrapie to humanized mice [4] or nonhuman primates [5,6].

Since 2002, EU adopted mandatory measures for the TSE surveillance on small ruminants to safeguard public and animal health. Each Member State (MS) has to carry out an annual monitoring program for TSE diseases based on active (testing on regularly slaughtered and risk animals) and passive (testing on clinical suspected cases) surveillances. More than 9 million small ruminants, 2/3 are sheep, have been tested as part of the official EU TSE surveillance (Reg. EU 999/2001) by one of the EU-approved tests and the number of tested animals in 2017 had an 8.7% (34,623 samples) increase compared with 2016.

TSEs are characterized by the misfolding of the host-encoded prion protein (PrP^c^) in its abnormal isoform (PrP^Sc^). The conformation of PrP^Sc^ differs from that of PrP^c^ in the amount of β-sheets leading to its tendency for aggregation and accounting for its partial proteinase K (PK) resistance [7]. PrP^Sc^ deposits and accumulates in great amount in the central nervous tissue (CNS) and, to a lesser extent, in peripheral tissues such as the lymphoreticular system (LRS) and other tissues or body fluids [8]. Most methods currently applied for the diagnosis of animal TSE diseases, including EU-approved ELISA tests and confirmatory western blots exploit the PK resistance of PrP^Sc^ and its great accumulation in the brain. Because the EU-approved tests need to be performed on CNS tissues, it is practically impossible to achieve a definite diagnosis of TSE disease in living animals. The detection of PrP^Sc^ in body fluids has been unsuccessful for a long time and, despite some recent promising results [9], the development of suitable tests for screening large numbers of animals is still lagging behind. Misfolded protein amplification techniques (Protein Misfolding Cyclic Amplification technology, PMCA, and Real-Time Quaking Induced Conversion assay, RT-QuIC) are able to amplify minute amounts of PrP^Sc^ in cerebrospinal fluid (CSF) and olfactory mucosa [9], and have been recently included in the international diagnostic criteria for sporadic CJD [10]. Such approaches, however, are of little utility for the screening of scrapie: lumbar puncture is a relatively invasive procedure and nasal brushing for taking the olfactory mucosa is impracticable in sheep for anatomical reasons. Finally, these tests are still not validated for the detection of PrP^Sc^ in blood despite that encouraging results have been obtained in some human and animal TSE diseases [11–14].

Recently, ultrasensitive immunoassay techniques such as single molecule array (Simoa, Quanterix, MA, USA) have enabled the reliable quantification of very low concentrations of proteins in body fluids [15], including neurofilament light (NfL) protein in blood samples [16]. NfL is a 68kDa cytoskeletal intermediate filament protein that is expressed in neuronal axons and released into the cerebrospinal fluid (CSF) and blood when neurons are injured [17], providing a valuable new biomarker with emerging diagnostic, prognostic, and therapy-monitoring roles in neurodegenerative and other human neurological diseases [17].

Here, we explore for the first time the value of serum NfL as marker for neuronal degeneration in sheep with natural scrapie disease. This is easily achievable because the amino acid sequence of the core domain of NfL, against which antibodies in the Simoa assay were developed, is 100% conserved in the animal kingdom [17].

## Materials and methods

### Animal cohort

The twenty Italian sheep included in the study were selected in the context of the scrapie surveillance system in Italy. Nine were field scrapie cases and 11 were healthy animals. Blood samples were collected just before slaughtering. The health status of the animals was assessed by the local veterinary service. Prion protein gene (*PRNP*) genotype defined on the basis of polymorphisms at codons 136, 154 and 171 was performed as described [18].

### Diagnosis of scrapie

Diagnosis of scrapie was performed on the *medulla oblongata* firstly by an enzyme-linked immunosorbent assay (ELISA)-based method (IDEXX HerdChek BSE-scrapie antigen test kit EIA rapid test—IDEXX Laboratories, Westbrook, ME, USA) followed by a confirmatory western blot method. The CEA internal western blot technique has been described elsewhere [19]. These methods represent the gold standard techniques for making the diagnosis of scrapie in SNC samples [20–21].

### NfL measurement

Serum NfL concentration in sheep sera was measured using an in house Simoa assay on an HD-1 Analyzer (Quanterix, Lexington, MA), as previously described in detail [16]. Board-certified laboratory technicians, blind to clinical and laboratory data, performed the measurements. Samples were measured in singlicates with a 4-fold dilution. Two quality control (QC) samples were analyzed in duplicates in the beginning and end of each run. For a QC sample with a concentration 11.3 pg/mL, repeatability was 4.0%. For a QC sample with a concentration 42.6 pg/mL, repeatability was 1.8%.

### Statistical analyses

Statistical analysis was performed using GraphPad Prism 5.0 software. Biomarker distributions were graphed as box plots and comparisons between groups were carried out by the non-parametric two-tailed unpaired Mann-Whitney *U* test with significance level of 5%. The efficiency of each biomarker was assessed by the receiver operating characteristic (ROC) curve analyses. Nonparametric ROC curves analyzed sheep with scrapie *vs* controls. The area under the ROC curve (AUC) and its 95% confidence interval (95% CI) indicate diagnostic efficiency. The accuracy of the test with the percent error is reported.

### Ethics statement

All animals used in this work were farm sheep slaughtered in official abattoirs and samples were taken in compliance with EU mandatory measures for the for the prevention, control and eradication of scrapie and other transmissible spongiform Encephalopathies (REGULATION (EC) No 999/2001, OJ L 147, 31.5.2001, p. 1).

## Results

The veterinary of the local health service reported that 2 of the 20 sheep used in this experiment showed clinical signs of scrapie consisting of proprioceptive deficit and low body condition score. The other 18 sheep were clinically unremarkable (Table 1). The ELISA test detected PrP^Sc^ in 9 sheep, including the 2 samples from animals with clinical signs. Western blot analysis confirmed the presence of PrP^Sc^ in all 9 samples that were positive in the ELISA test. The other 11 sheep were negative at both the preliminary ELISA test and the confirmatory western blotting analysis. There was no significant difference (p = 0.5913; Fisher’s exact test) in the M/F distribution between scrapie (1/8) and control (3/8) sheep. All sheep carried the ARQ/ARQ genotype of the prion protein gene.

**Table 1:** Serum neurofilamen light (NfL) in sheep with scrapie and controls.

Group of animals (n)	Neurological signs	Serum NfL (pg/mL)	Median (IQR)
Scrapie (9)	NO	116.5	56.2 (42.2–84.8)
		55.9	
		35.5	
		46.8	
		56.2	
		78.7	
		37.5	
	YES	90.9	
		59.6	
Controls (11)	NO	2.5	3.4 (3.0–26.3)
		3.1	
		26.5	
		3.0	
		3.4	
		3.4	
		3.1	
		2.7	
		56.4	
		26.3	
		8.4	

Wilcoxon-Mann-Whitney U test, p = 0.00084

**Fig 1:**
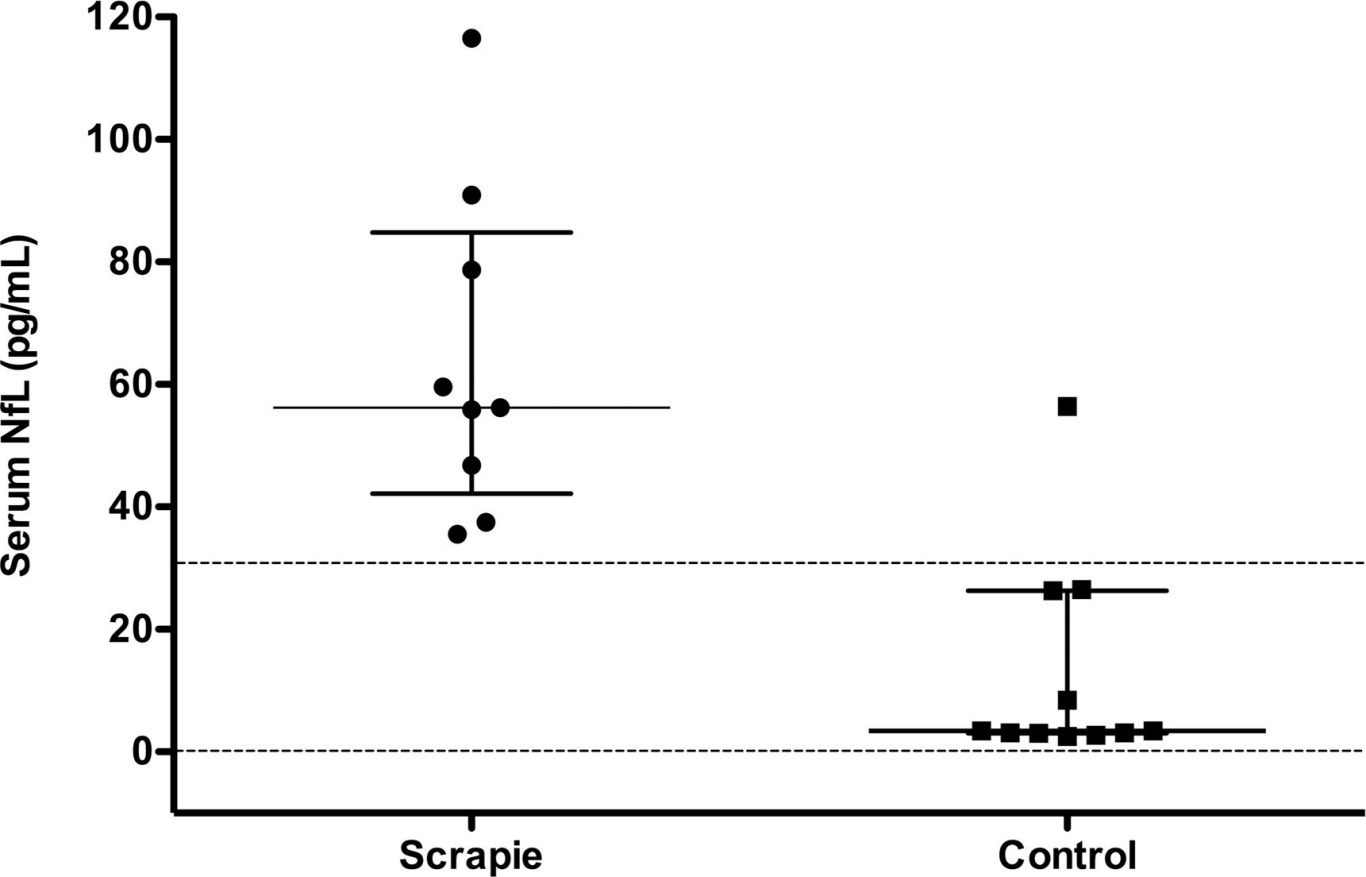
Concentrations of serum NfL in scrapie-affected sheep (circles) and controls (squares). The central bars indicate median values; upper and bottom bars indicate interquartile ranges. The top dashed line is the cut-off value of 31 pg/ml of serum NfL obtained from the ROC curve analyses (Fig 2) that gives 90.9% specificity and 100% sensitivity, while the bottom dashed line indicates the lowest limit of detection (LLOD) of NfL (0.62 pg/ml).

**Fig 2:**
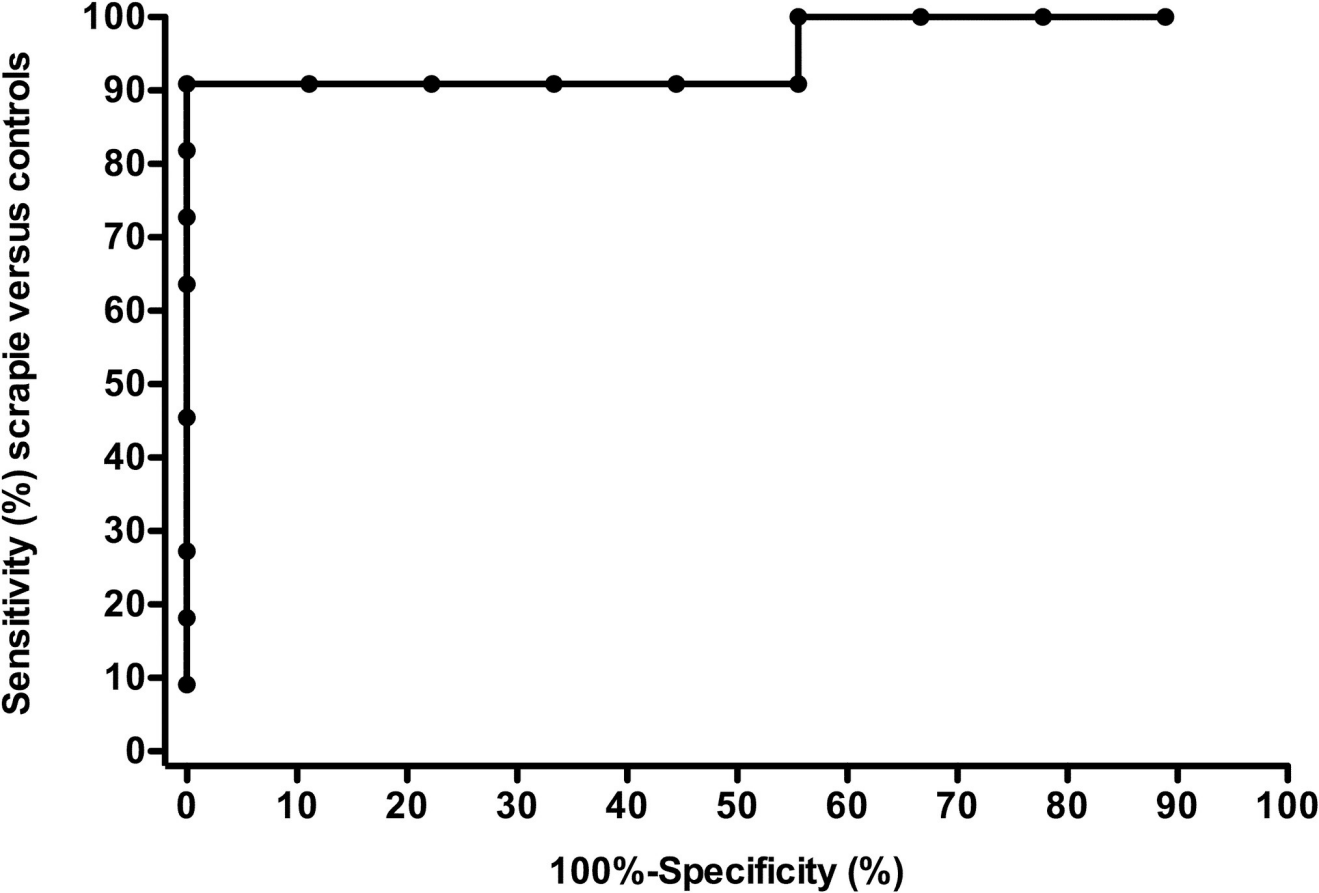
Receiver operating characteristic (ROC) curve analysis. The nonparametric ROC curve for NfL in the comparative analysis for scrapie-affected sheep versus controls. Area under the curve (95% CI) = 0.9496 (0.8467 to 1.052); p = 0.0007.

The concentration of serum NfL in each sample is shown in Table 1. The median serum NfL concentration in scrapie animals was more than 15 times higher than that found in control samples (Table 1). However, the serum NfL concentration in scrapie sheep with clinical signs (n = 2; 75.3, 15.7 pg/ml) did not significantly (p = 0.541; t-test) differ from scrapie animals without clinical signs (n = 7; 61.0, 10.7 pg/ml). It is of note that all control animals but one had serum NfL concentration well below the lowest level of scrapie sheep (Table 1 and Fig 1) suggesting that this test is valuable for making an accurate screening of scrapie in living sheep The ROC curve (Table 1 and Fig 2) provided the cut-off value of 31 pg/ml serum NfL for distinguishing scrapie from control sheep with an accuracy of the test of 95% (percent error, 5.26%). Considering only animals with no clinical signs of scrapie (n = 18), the accuracy of the test for the preclinical identification of scrapie-infected sheep was 94.4% (percent error, 5.9%).

## Discussion

Our data show that scrapie-affected sheep have significantly higher levels of serum NfL than control sheep, similarly to what has been reported for human TSEs [22–25], other patients affected by degenerative or inflammatory diseases of the nervous system [17], and experimental animal models of neurodegenerative diseases [26–29]. These results confirm the importance of the Simoa test to quantify small amount of NfL in blood and other body fluids and the validity of NfL as a sensitive biomarker for neuronal and axonal damage [17]. However, serum NfL show low specificity in distinguishing CJD from other neurodegenerative diseases [24], and are therefore of limited value for making a differential diagnosis in living patients [26]. Although CSF Nfl is able to distinguish CJD from other neurodegenerative dementias with fairly good accuracy (AUC ≈0.90) [30,31], in suspected CJD patients, CSF samples are routinely obtained for confirming or excluding diagnosis with the highly specific and sensitive RT-QuIC test for prion [9]. Contrary to what occurs in humans where CJD is an extraordinarily rare disease [32] compared with other neurodegenerative diseases, scrapie is one of the most common neurological diseases in sheep [33,34], and veterinarians usually do not perform spinal puncture in sheep or other farm animals and lack sophisticated diagnostic tools, such as brain MRI and PET scans. Thus, the use of the Simoa test for measuring serum NfL makes progress in the diagnosis of scrapie in living sheep. Moreover, the finding that blood samples of asymptomatic scrapie-affected sheep had serum NfL levels similar to those observed in clinically affected sheep suggests that serum NfL increases very early in the disease process and that the Simoa test is a quicker and more efficient tool for the preclinical screening of asymptomatic sheep in flocks at risk for scrapie than 14-3-3 and tau CSF biomarkers [35]. Our results are in agreement with recent findings showing that in genetic Alzheimer disease [36] and frontotemporal dementia [37] the level of serum NfL increase in mutation carriers before symptom onset. It would be interesting to compare the concentration of NfL in the serum of prion-affected individuals with the amount of PrP^Sc^ in the CNS for determining any eventual correlation.

The preliminary data reported here need, however, to be confirmed in large and more structured studies before the introduction of the Simoa test for measuring serum NfL in sheep. Comparing scrapie with healthy controls likely overestimated the diagnostic accuracy reported in this study and more appropriate field studies with a large cohort of animals affected by various pathologies mimicking scrapie (e.g. coenurosis, leptospirosis, etc.) are needed for a better estimate of sensitivity and specificity in recognizing scrapie disease. The scrapie negative sample (Table 1 and Fig 1) with high serum NfL concentration was an unexpected result although it might be related to neuroaxonal injury in asymptomatic or preclinical inflammatory, infectious, or traumatic neurological conditions [38]. Finally, in this study all samples were taken from sheep carrying the same *PRNP* genotype (ARQ/ARQ) and it is critical to extend this analysis to blood samples taken from other genotypes. It is well established that *PRNP* genotypes in sheep influence susceptibility to scrapie and likely determine different clinical and pathological phenotypes [39].

In conclusion, we believe that the improved sensitivity of the digital immunoassay (Simoa) over previously reported approaches will translate into preclinical diagnostic benefits of paramount importance for managing scrapie disease in sheep and possibly other animal prion diseases.
